# Continued circulation of mpox: an epidemiological and phylogenetic assessment, European Region, 2023 to 2024

**DOI:** 10.2807/1560-7917.ES.2024.29.27.2400330

**Published:** 2024-07-04

**Authors:** Aisling M Vaughan, Mohammed Afzal, Priyanka Nannapaneni, Mathias Leroy, Xanthi Andrianou, Jeffrey Pires, Silvia Funke, Celine Roman, Juliana Reyes-Uruena, Stephan Aberle, Aristos Aristodimou, Gudrun Aspelund, Kirsty F Bennet, Antra Bormane, Anna Caraglia, Hannah Charles, Emilie Chazelle, Iva Christova, Orna Cohen, Costas Constantinou, Simon Couvreur, Asuncion Diaz, Kateřina Fabiánová, Federica Ferraro, Marte Petrikke Grenersen, Eva Grilc, Tuula Hannila-Handelberg, Anne Kathrine Hvass, Derval Igoe, Klaus Jansen, Denisa Janță, Styliani Kaoustou, Anders Koch, Mirjana Lana Kosanovic Licina, Stefka Krumova, Anton Labutin, Raskit Lachmann, Amaryl Lecompte, Rémi Lefrançois, Viktorija Leitena, Kirsi Liitsola, Ivan Mlinarić, Zohar Mor, Martha Neary, Alina Novacek, Magnus Wenstøp Øgle, Hana Orlíková, Kalliopi Papadima, Moa Rehn, Malgorzata Sadkowska-Todys, Anca Sîrbu, Klara Sondén, Berta Suárez, Marianna Thordardottir, Paula Vasconcelos, Joao Vieira Martins, Karolina Zakrzewska, Marc-Alain Widdowson, Céline M Gossner

**Affiliations:** 1 World Health Organization (WHO) Regional Office for Europe, Copenhagen, Denmark; 2 European Centre for Disease Prevention and Control (ECDC), Stockholm, Sweden; 3 Medical University of Vienna, Centre for Virology, Vienna, Austria; 4 Medical and Public Health Services, Nicosia, Cyprus; 5 Centre for Health Security and Communicable Disease Control, Directorate of Health, Reykjavik, Iceland; 6 United Kingdom Health Security Agency, London, United Kingdom; 7 Center for Disease Prevention and Control of Latvia, Department of Infectious Diseases Risk Analysis and Prevention, Riga, Latvia; 8 Ministry of Health, Rome, Italy; 9 Santé publique France, the French National Public Health Agency, Saint-Maurice, France; 10 National Center of Infectious and Parasitic Diseases, Sofia, Bulgaria; 11 Division of Epidemiology, Ministry of Health, Jerusalem, Israel; 12 Department of Epidemiology and Public Health, Sciensano, Brussels, Belgium; 13 National Centre of Epidemiology, CIBER in Infectious Diseases (CIBERINFEC), Carlos III Health Institute, Madrid, Spain; 14 National Institute of Public Health, Centre for Epidemiology and Microbiology, Department of Infectious Disease Epidemiology, Prague, Czechia; 15 Norwegian Institute of Public Health (NIPH), Oslo, Norway; 16 NIJZ (NIPH), Centre for Communicable Diseases, Ljubljana, Slovenia; 17 Finnish Institute for Health and Welfare, Helsinki, Finland; 18 Department of Infectious Disease Epidemiology and Prevention, Statens Serum Institut, Copenhagen, Denmark; 19 HSE Public Health: National Health Protection Office, Dublin, Ireland; 20 Department for Infectious Disease Epidemiology, Robert Koch Institute, Berlin, Germany; 21 National Institute of Public Health, Bucharest, Romania; 22 Directorate of Epidemiological Surveillance and Intervention for Communicable Diseases, National Public Health Organization, Marousi, Greece; 23 Department of Infectious Diseases, Rigshospitalet University Hospital, Copenhagen, Denmark; 24 Andrija Stampar Teaching Institute of Public Health, Zagreb, Croatia; 25 Swiss Federal Office of Public Health, Bern, Switzerland; 26 Santé publique France, the French National Public Health Agency, Saint-Denis, France; 27 Croatian Institute of Public Health, Division for Epidemiology of Communicable Diseases, Zagreb, Croatia; 28 School of Health Sciences, Ashkelon Academic College, Ashkelon, Israel; 29 HSE Health Protection Surveillance Centre, Dublin, Ireland; 30 Austrian Agency for Health and Food Safety, Vienna, Austria; 31 Department of Epidemiology and Biostatistics, Third Faculty of Medicine, Charles University, Prague, Czechia; 32 Public Health Agency of Sweden (PHAS), Solna, Sweden; 33 National Institute of Public Health NIH - National Research Institute, Warsaw, Poland; 34 Coordinating Centre for Health Alerts and Emergencies (CCAES), Directorate General of Public Health, Ministry of Health, Madrid, Spain; 35 Public Health Emergency Centre, Directorate-General of Health (DGS), Lisbon, Portugal; 36 Directorate of Information and Analysis, Directorate-General of Health, Lisbon, Portugal; * These authors contributed equally as first authors; ** These authors contributed equally as last authors

**Keywords:** MPXV, Mpox, outbreak, orthopoxvirus, WHO European Region, Europe

## Abstract

During the summer of 2023, the European Region experienced a limited resurgence of mpox cases following the substantial outbreak in 2022. This increase was characterised by asynchronous and bimodal increases, with countries experiencing peaks at different times. The demographic profile of cases during the resurgence was largely consistent with those reported previously. All available sequences from the European Region belonged to clade IIb. Sustained efforts are crucial to control and eventually eliminate mpox in the European Region.

Since 2022, the World Health Organization (WHO) European Region has been experiencing an outbreak of mpox, predominantly affecting men who have sex with men (MSM) [1]. The first cases were retrospectively detected in March 2022, reaching a peak in July of the same year. Subsequently, case numbers declined rapidly and remained at low levels, until a limited increase started from June 2023. Here, we provide an overview of the upsurge in cases in the Region using data up to 10 June 2024 and highlight key public health measures.

## Data sources and analysis

Data on mpox cases in the WHO European Region are reported to the European Centre for Disease Prevention and Control (ECDC) and the WHO via the European Surveillance System (TESSy; hosted by the ECDC), in line with the WHO Standing Recommendations [2]. Cases were reported following WHO, ECDC or national case definitions valid at the time. We examined two distinct timeframes of monkeypox virus (MPXV) activity since the first case in the Region: Period 1 covered week 10 of 2022 (first mpox case detected in the European Region and reported to TESSy) to week 22 of 2023 (lowest 3 week moving average value); Period 2 covered week 23 of 2023 to week 21 of 2024.

We performed a descriptive analysis of cases and used a Pearson’s chi-square test for comparisons between periods. All analyses were performed in R software version 4.3.0. We performed phylogenetic analysis on publicly available MPXV sequences from NCBI GenBank using Nextstrain build (https://github.com/nextstrain/mpox).

## Epidemiological situation

Since May 2022, 27,298 mpox cases have been reported in the WHO European Region, of which 22,796 (84%) were reported by countries in the European Union/European Economic Area (EU/EEA). After the peak in July 2022, cases rapidly declined and remained low until a mild resurgence from June 2023. The number of cases increased from approximately 6.6 weekly cases in the first 5 months of 2023 to 30.3 weekly cases during the rest of the year. Overall, the numbers of cases reported in Period 2 (n = 1,432) were far fewer than in Period 1 (n = 25,866), with a peak of 2,714 cases reported in week 27 of 2022 (Period 1) and 54 cases reported in week 39 of 2023 (Period 2). In addition, the number of affected countries decreased from 41 countries reporting between one and 7,571 cases in Period 1, to 25 countries reporting between one and 459 cases in Period 2. Cases reported before August 2022 are described in further detail elsewhere [1].

**Figure 1:**
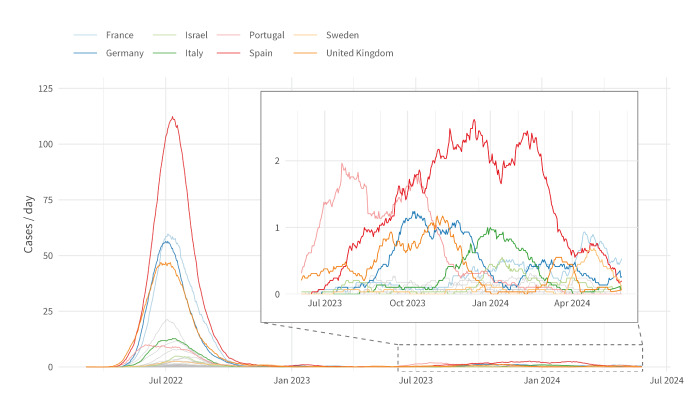
30-day centred moving average of daily mpox cases, by country, 2022–2024 (n = 27,298)

Of the 25 countries that reported cases in Period 2, five – Spain, Portugal, Germany, the United Kingdom (UK) and France – experienced a notable resurgence, accounting for 76% (1,095/1,432) of cases reported in Period 2, while these countries also contributed heavily to the cases in Period 1, accounting for 78% (20,064/25,866) of cases. This increase was largely asynchronous and bimodal, and countries experienced peaks at different times. In the UK, cases rose steadily from nine cases in May 2023 to a peak of 31 cases in November 2023, while in Portugal, cases rose from 18 in June 2023 to a peak of 50 in October 2023. Similarly, cases in Spain increased from nine in July 2023 to a peak of 76 cases in December 2023. In Germany and France, the numbers also increased, starting from August 2023 and November 2023, respectively. More recently, since March 2024, France, Sweden, Germany and the UK have experienced a slight rise after a period of low transmission. In the other countries, low levels of mpox were reported throughout the period. (Figure 1).

30-day centred moving average of daily number of mpox cases, by date of symptom onset or earliest date of diagnosis or notification if missing. The box displays cases per day for the period between W23/2023 to W21/2024, defined as Period 2 in this study. Countries with increase in cases in Period 2 are highlighted. The grey line represents the rest of the countries in the World Health Organization European Region.

**Figure 2:**
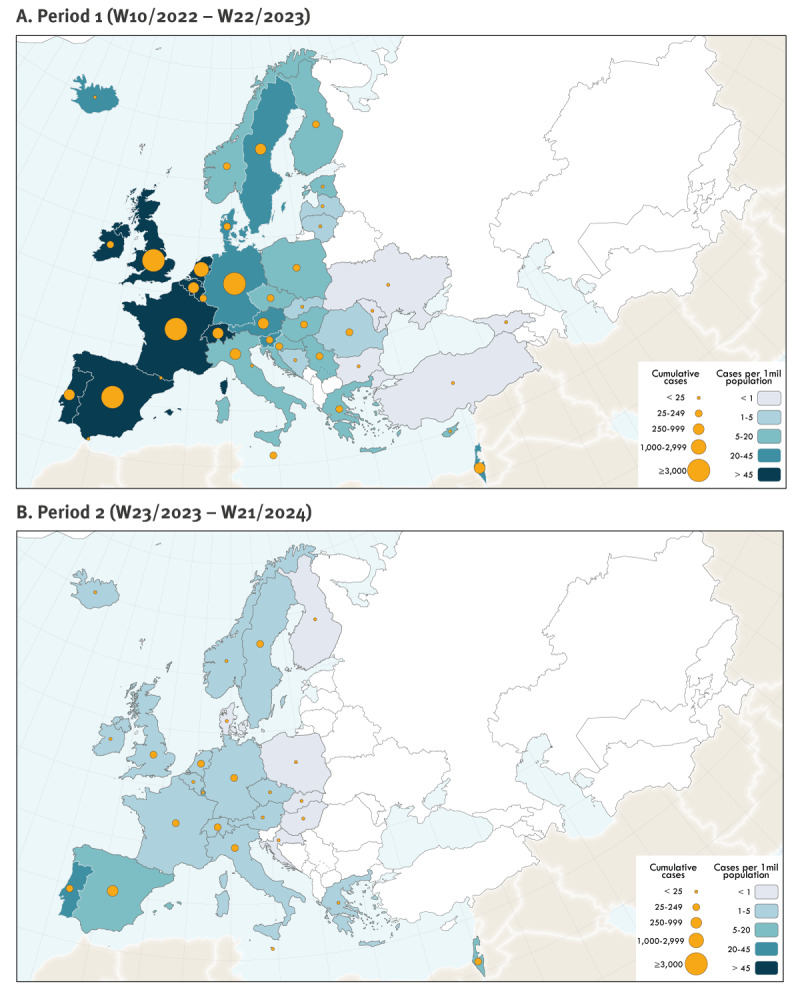
Geographical distribution of reported mpox cases, WHO European Region, 2022–2024 (n = 27,298)

In Period 1, Spain experienced the most substantial epidemic in the Region (n = 7,571 cases) followed by France (n = 4,147), the UK (n = 3,709), Germany (n = 3,683), the Netherlands (n = 1,266), Italy (n = 959) and Portugal (n = 954). A further 34 countries reported a range between one and 794 cases. In Period 2, Spain reported most cases (n = 459), followed by Portugal (n = 239), Germany (n = 156) and France (n = 102). While the Netherlands experienced a notable surge in 2022, they have not experienced a resurgence in Period 2, reporting 36 cases until 10 June 2024 (Figure 1, Figure 2). In Supplementary Figure S1 we provide epidemic curves for 10 countries with the highest cumulative cases reported since May 2022.

WHO: World Health Organization.

White: Countries or areas in WHO European Region with no reported cases. Brown: Countries and areas outside the WHO European Region.

## Demographic characteristics

 The case demographics in Period 2 (n = 1,432) and Period 1 (n = 25,866) were broadly similar; of cases where information was available, the majority were male (99% in Period 2 vs 98% in Period 1), MSM (96% vs 96%) and 31–40 years-old (39% vs 39%). Cases in Period 2 were significantly younger than in Period 1 (18–30 years: 29% vs 25%; 41–50 years: 22% vs 24%; 51–60 years: 8% vs 9%; p < 0.05). Where information was available, sexual transmission was reported as the main mode of transmission (97% vs 95%). Of 574 cases with non-sexual modes of transmission, person-to-person transmission excluding sexual transmission was reported for 537 (94%) cases, contact with contaminated material for 32 (6%), hospital-associated transmission for four (< 1%), and transmission in a laboratory due to occupational exposure in one (< 1%).

Among cases with known HIV status (n = 11,466), 38% (4,330) were among people living with HIV and this remained stable over time. Disease severity also remained stable over time, with hospitalisation rates between 5% and 6% across periods. In total, eight individuals were admitted to intensive care units and 10 cases died (Table). Of the 10 cases who died, information on HIV status was known for nine cases, eight of whom were living with HIV.

**Table:** Epidemiological and clinical characteristics of reported mpox cases in Europe: comparison between the two defined periods of increased mpox activity, 2022–2024 (n = 27,298)

	Overall cases (n = 27,298)		Period 1 (n = 25,866)		Period 2 (n = 1,432)		p value^a^
			W10/2022–W22/2023		W23/2023–W21/2024		
	n	%	n	%	n	%	
Age group (years)							
0–14	35	0.1	33	0.1	2	0.1	0.0190
15–17	58	0.2	54	0.2	4	0.3	
18–30	6,961	25.5	6,541	25.3	420	29.4	
31–40	10,741	39.4	10,190	39.4	551	38.6	
41–50	6,435	23.6	6,124	23.7	311	21.8	
51–60	2,431	8.9	2,324	9.0	107	7.5	
> 60	602	2.2	568	2.2	34	2.4	
Unknown	35	NC	32	NC	3	NC	NC
Median age (IQR)	36 (30–43)		36 (30–44)		35 (29–43)		NC
Gender^b^							
Male	26,781	98.3	25,371	98.3	1,410	98.7	0.020
Female	448	1.6	432	1.7	16	1.1	
Other	17	0.1	14	0.1	3	0.2	
Unknown	52	NC	49	NC	3	NC	NC
Sexual behaviour							
Men who have sex with men	12,084	95.5	11,377	95.5	707	95.9	NC
Heterosexual	518	4.1	492	4.1	26	3.5	
Bisexual	53	0.4	49	0.4	4	0.5	
Women who have sex with women	0	0.0	0	0.0	0	0.0	
Unknown	14,643	NC	13,948	NC	695	NC	
Mode of transmission							
Sexual^c^	10,609	94.9	9791	94.7	818	97.3	0.001
Non-sexual	574	5.1	551	5.3	23	2.7	
Unknown	16,115	NC	15,524	NC	591	NC	NC
Travel^d^							
Yes	2,264	20.4	2,091	20.5	173	19.4	0.425
No	8,813	79.6	8,092	79.5	721	80.6	
Unknown	16,221	NC	15,683	NC	538	NC	NC
Severity							
Hospitalised^e^	690	5.3	634	5.3	56	6.0	NC
Intensive care admission^f^	8	0.1	8	0.1	0	0.0	
Death^g^	10	0.1	7	0.0	3	0.3	
HIV status							
Positive	4,330	37.8	4,057	38.0	273	34.8	0.080
Negative	7,136	62.2	6,624	62.0	512	65.2	
Unknown	15,832	NC	15,185	NC	647	NC	NC
Historical smallpox vaccination							
Vaccinated	1,152	14.2	1,112	14.7	40	7.3	< 0.001
Not vaccinated	6,944	85.8	6,433	85.3	511	92.7	
Unknown	19,202	NC	18,321	NC	881	NC	NC
Vaccination for current event^h^							
Vaccinated (PPV, PEPV, vaccination strategy unknown)	438 (126, 71, 241)	12.6 (3.6, 2.0, 7.0)	284 (73, 67, 144)	9.1 (2.3, 2.1, 4.6)	154 (53, 4, 97)	44.6 (15.4, 1.2, 28.1)	< 0.001
Not vaccinated	3,026	87.4	2,835	90.9	191	55.4	
Unknown	23,834	NC	22,747	NC	1,087	NC	NC

NC: not calculated; PEPV: post-exposure preventive vaccination; PPV: primary preventive (pre-exposure) vaccination.

^a^ Pearson’s chi-squared test p values of < 0.05 were considered statistically significant.

^b ^Gender collected in TESSy as female, male, other (e.g. transgender) or unknown.

^c ^One case reported two modes of transmission (sexual and person-to-person). Non-sexual transmission includes person-to-person transmission (excluding sexual transmission), healthcare-associated transmission, laboratory occupational exposure and contact with contaminated material.

^d^ Travel history outside the country of notification during the incubation period.

^e ^Includes cases hospitalised for treatment and unknown reason. Isolation not included in the hospitalisation (overall hospitalised for isolation: n = 193; 1.5%). The denominator used for calculating the percentages were the sum of the cases that were reported to be hospitalised for treatment, hospitalised for unknown reason, hospitalised for isolation and not hospitalised.

^f^ The denominator used for calculating the percentages was the sum of the cases that were reported to be admitted to intensive case and that were reported not admitted to intensive care.

^g ^The denominator used for calculating the percentages was the sum of the cases that were reported as alive and dead.

^h ^Relates to vaccination since 2022.

The proportion of cases who reported historical smallpox vaccination during routine smallpox vaccination programmes was significantly lower in Period 2 compared with Period 1 (7% vs 15%; p < 0.001). The proportion of cases vaccinated against mpox since 2022 was fivefold higher in Period 2 (45% vs 9%), however, information on vaccination was available for a larger proportion of cases in Period 2 (24% vs 12%). The proportion of cases who received primary preventive (pre-exposure) vaccination (PPV) increased approximately sevenfold (15% vs 2%), while the proportion of cases who received post-exposure preventive vaccination (PEPV) did not differ substantially between the two periods (1% vs 2%) (Table). Overall, 87% (n = 3,026) of cases had not received vaccination since 2022, a reduction from 91% (n = 2,835) of all cases with available information in Period 1 to 55% (n = 191) in Period 2.

The majority of cases were due to locally acquired infection, and the overall proportion of cases who reported travel within 21 days before disease onset remained largely constant over time (21% vs 19%), however, differences were observed between countries. In Supplementary Table S1 we append further detail on travel history by country. During Period 1, Spain (n = 446; 33%), Germany (n = 161; 12%) and France (n = 125; 9%) were the most visited destinations among travel-related cases, and during Period 2, Spain (n = 27; 29%), the UK (n = 7; 8%), Germany (n = 6; 6%) and France (n = 6; 6%) were the most visited.

## Molecular characteristics of monkeypox virus

**Figure 3:**
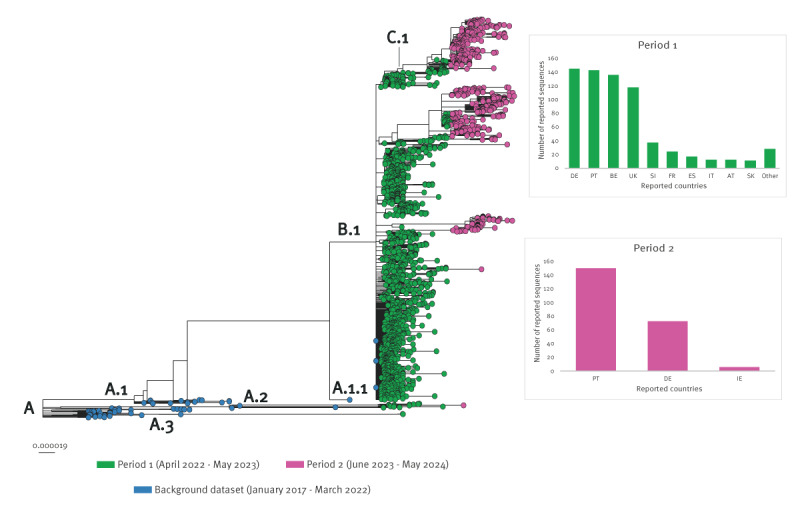
Phylogenetic analysis of clade IIb monkeypox virus publicly available sequences from NCBI, 2017–2024 (n = 1,943)

Of the 2,009 MPXV sequences analysed globally, 1,349 sequences (67%) were collected during Period 1, and 571 (28%) in Period 2, with variation in the proportion of sequences available by countries. Of the 2,009 sequences, 922 sequences were from the European Region, 683 (74%) collected in Period 1 and 229 (25%) collected in Period 2. The majority of global sequences (n = 1,943; 97%) belonged to MPXV clade IIb, while 13 sequences were Clade IIa and the remaining sequences belong to Clade I (Figure 3). The clade lla and clade I sequences were from outside the European Region. Clade IIb sequences were dominated by the B.1 lineage and did not cluster by geographical region, indicating community transmission both within and across the regions due to importations. In Period 2, sequences clustered into distinct major clusters (some country-specific clusters) belonging to B.1 and C.1 lineages. Overall, Period 2 isolates had longer branch lengths than Period 1 isolates, indicating ongoing virus evolution with an accumulation of adaptative mutations over time (Figure 3).

AT: Austria, BE: Belgium, DE: Germany, ES: Spain, FR: France, IE: Ireland, IT: Italy, PT: Portugal, SI: Slovenia, SK: Slovakia, UK: United Kingdom.

Viral sequences from 2017 to 2024 were assigned to a specific clade using Nextclade and filtered for the Nextstrain curated exclusions, minimum length of 100,000 bp, collection time from 2017 onwards and subsampling to a maximum of 2,000 samples per country during the same sampling month. IQTREE was used to construct the tree, TreeTime to refine the tree and visualized using MicroReact. Only Clade IIb isolates are shown in the figure. Isolates are displayed as well as coloured in the tree by reporting period. Period 1 isolates collected during April 2022 to May 2023 are coloured in green, Period 2 isolates collected during June 2023 to May 2024 are coloured in red. Sequences outside the collection periods of this study are labelled as background dataset and these are from collection period January 2017 to March 2022 and coloured in blue.

Bar charts depict coverage from countries in the European Region for both periods. Countries that reported fewer than 10 sequences in Period 1 include Czechia, Finland, Ireland, Israel, the Netherlands, Romania, Sweden and Switzerland. The MicroReact link to visualise data and genomic epidemiology of the sequences including clade I, IIa and IIb isolates is available athttps://microreact.org/project/jvF1komkH15f1sTcJGsn8s-20240627-mpox-phylogeny.

## Discussion

Starting in the summer of 2023, the number or mpox cases in the WHO European Region increased, although the numbers were markedly smaller than during the major outbreak in the previous year. Cases continue to be reported at low levels, characterised by a predominance of sexual transmission among MSM, and a slight shift to younger age groups was noted.

The decline and subsequent resurgence are not yet fully understood but may result from behavioural changes and/or acquired immunity due to vaccination or prior infection in at-risk groups [3-7]. Further investigation is needed to inform effective national preparedness and control strategies going forward.

Notably, a disproportionate number of cases in the Region were people living with HIV, and almost all of the 10 cases who died were reported to be HIV-positive. While those living with controlled HIV appear not to be at higher risk of severe mpox disease, evidence suggests that those with undiagnosed or uncontrolled HIV have worse clinical outcomes [8]. Therefore, it is important to ensure prevention and clinical care for those at risk of severe mpox disease, ensuring individuals with mpox and unknown HIV status are offered HIV testing and those living with HIV are tested for MPXV when clinically indicated. Furthermore, targeted intervention is essential for higher-risk individuals, such as those using in HIV pre-exposure prophylaxis and sexually transmitted infection services, marginalised populations and other vulnerable groups.

The higher proportion of cases historically vaccinated against smallpox in Period 1 could partially be explained by the older age of cases in this period, as routine smallpox vaccination programmes ended by the 1980s. These data require careful interpretation as available vaccination information was limited and data were available for a much higher proportion of cases in the second period. In Period 2, a larger proportion of cases were vaccinated against mpox, likely related to increased vaccine availability and heightened awareness among high-risk groups. Available evidence suggests that vaccination against smallpox protects against mpox [9-14] and so remains a critical component of the mpox response. However, a notable proportion of cases remained unvaccinated, underscoring the need for ongoing efforts to improve vaccine availability and coverage for key populations at higher risk of exposure and infection.

All available sequences from the European Region belong to clade IIb, with no evidence of the more severe clade I virus circulation. However, the geographical expansion of mpox in other regions and sexual transmission of clade I in endemic countries is of concern [14,15], and future transmission in the European Region cannot be excluded. Robust laboratory-based surveillance, rapid detection and broadly available genomic sequencing will support the detection of a potential emergence of clade I in the European Region.

A limitation of this study was the incompleteness of several variables and that analyses were performed on surveillance data reported by countries, which vary in completeness and availability, and may be subject to reporting delays and to reporting or diagnostic biases. Similarly, the phylogenetic analyses were constrained by limited sequence availability, therefore strengthened sequencing capacities are needed to support a more comprehensive understanding of the evolution of MPXV in the European Region. The geographical bias towards the west of the Region is not fully understood but may be due to varying sexual behaviours among different MSM communities, transmission dynamics, or limited testing and surveillance capacities, lack of awareness or limited healthcare access for potentially stigmatised groups.

According to the classification in WHO Regional office for Europe’s regional control and elimination strategy [16], 46 of the 62 countries and areas in the Region are considered as level 1a (countries/areas that have not yet detected a case of mpox or have not detected a case for 3 months or more in the presence of quality surveillance), three are at level 1b (countries/areas with imported or travel-related case(s) of mpox in the human population with onset in the previous 3 months) whereas 13 remain as level 2 (sustained local human-to-human transmission with locally acquired infection in last 3 months). Of those at level 2, five countries reported fewer than five cases in last 3 months. The national transmission classifications outlined here are based solely on surveillance data, the detection rates of which may vary among different population. Further metrics are required to effectively monitor and assess the epidemiological situation and countries’ progress towards control and elimination of human-to-human transmission as outlined in the WHO Strategic Framework for Enhancing Prevention and Control of mpox 2024–2027 [17,18].

## Conclusion

While concerted efforts by countries, communities and stakeholders have been successful in substantially reducing the incidence of mpox in Europe, MPXV continues to spread at low levels and therefore continues to pose a risk to affected populations. As we have reached the summer period, potential transmission-amplifying events such as pride and circuit festivals may further increase cases, therefore it is important that countries continue their efforts to successfully control human-to-human transmission and mitigate any future resurgences. Such efforts should include prioritising testing; integration of mpox prevention, screening, treatment and reporting into existing health programmes and services; ensuring vaccine accessibility for individuals at high-risk; enhancing risk communication for widespread awareness, mitigating stigma and misinformation, and fostering community engagement to promote awareness of and adherence to risk reduction strategies. Continued efforts in these areas are crucial to control and eventually eliminate mpox in the European Region.

## Supplementary Data

353000 Supplement
