# Supplementary material for: Continued circulation of mpox: an epidemiological and phylogenetic assessment, European Region, 2023 to 2024
Source: Euro Surveill. 2024 Jul 4;29(27):2400330. doi: 10.2807/1560-7917.ES.2024.29.27.2400330 (PMC11225264; doi:10.2807/1560-7917.ES.2024.29.27.2400330)
Supplement: Supplement [file 24-00330_VAUGHAN_Supplement.pdf]

Supplementary Figures and Tables

Disclaimer: This supplementary material is hosted by *Eurosurveillance* as supporting information alongside the article **'Continued circulation of mpox: an epidemiological and phylogenetic assessment, European Region, 2023 to 2024'**, on behalf of the authors, who remain responsible for the accuracy and appropriateness of the content. The same standards for ethics, copyright, attributions and permissions as for the article apply. Supplements are not edited by *Eurosurveillance* and the journal is not responsible for the maintenance of any links or email addresses provided therein.

Supplementary Figure S1: Number of confirmed and probable cases reported in the European region, 2022–2024 (n= 27,298)

a) Epidemic curve of reported mpox cases since May 2022 b) Individual epidemic curves for ten countries with the highest cumulative cases reported since May 2022. Figure legend: Distribution of reported cases by symptom onset or earliest date of diagnosis, or notification if missing.

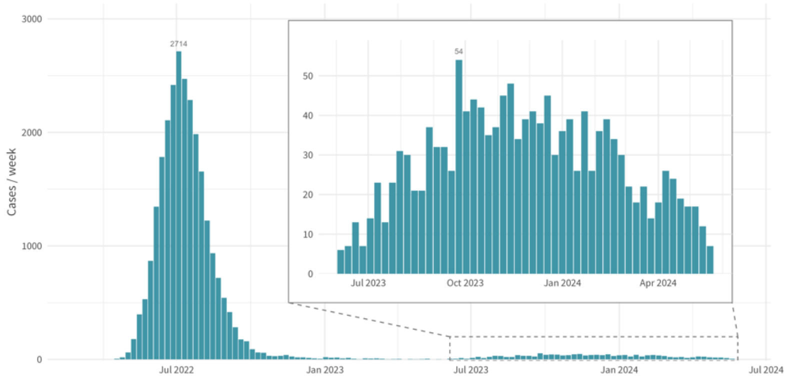

a)

ECDC NORMAL

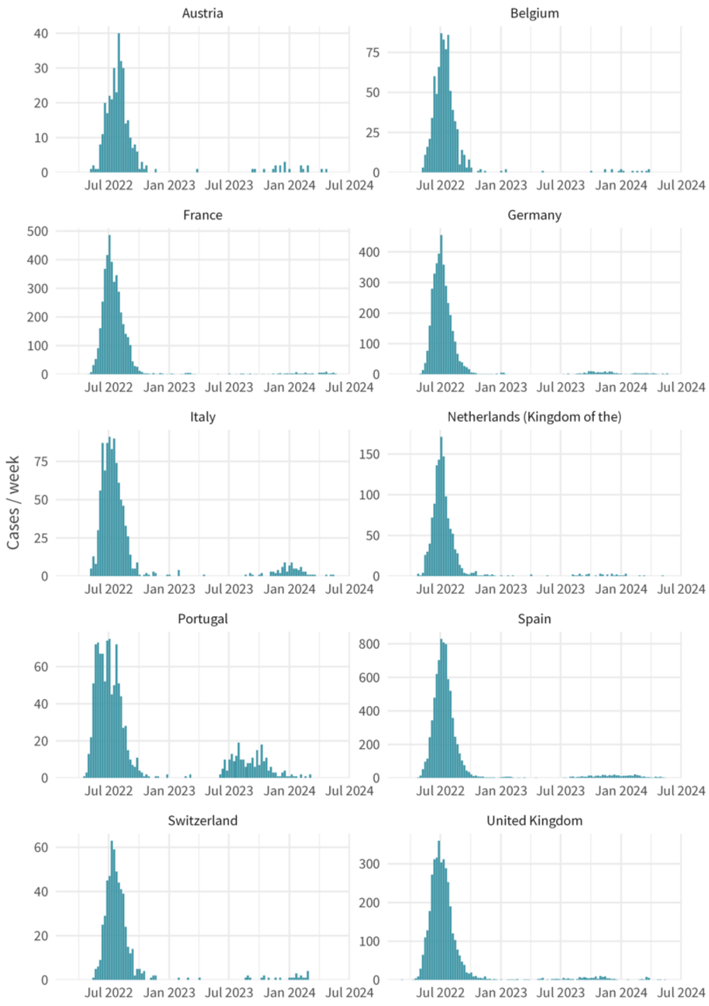

b)

Supplementary Table S1: Travel history of cases reported in period 1 and period 2

Country	Period 1				Period 2			
	Total	Travel history[a]			Total	Travel history[a]		
		Yes	No	Unknown		Yes	No	Unknown
Austria	328	68 (28.2%)	173 (71.8%)	87	19	4 (25%)	12 (75%)	3
Belgium	794	242 (35.5%)	440 (64.5%)	112	15	4 (28.6%)	10 (71.4%)	1
Croatia	33	4 (12.1%)	29 (87.9%)	0	1	0 (0%)	1 (100%)	0
Czechia	71	21 (29.6%)	50 (70.4%)	0	11	2 (18.2%)	9 (81.8%)	0
Denmark	196	57 (29.7%)	135 (70.3%)	4	2	0 (0%)	2 (100%)	0
Finland	42	0	0	42	1	0	0	1
France	4147	0	0	4147	102	0	0	102
Germany	3683	0	0	3683	156	0	0	156
Greece	88	22 (25.3%)	65 (74.7%)	1	11	1 (9.1%)	10 (90.9%)	0
Hungary	80	24 (30.4%)	55 (69.6%)	1	3	1 (33.3%)	2 (66.7%)	0
Iceland	16	7 (43.8%)	9 (56.3%)	0	1	1 (100%)	0 (0%)	0
Ireland	232	118 (61.1%)	75 (38.9%)	39	15	4 (33.3%)	8 (66.7%)	3
Israel	264	23 (51.1%)	22 (48.9%)	219	48	8 (19%)	34 (81%)	6
Italy	959	0	0	959	88	0	0	88
Luxembourg	57	26 (45.6%)	31 (54.4%)	0	4	4 (100%)	0 (0%)	0
Malta	34	15 (44.1%)	19 (55.9%)	0	1	1 (100%)	0 (0%)	0
Netherlands (Kingdom of the)	1266	0	0	1266	36	0	0	36
Norway	95	45 (48.9%)	47 (51.1%)	3	11	5 (50%)	5 (50%)	1
Poland	217	41 (20.4%)	160 (79.6%)	16	6	3 (50%)	3 (50%)	0
Portugal	954	125 (18.6%)	548 (81.4%)	281	239	22 (10.6%)	185 (89.4%)	32

Slovakia	14	6 (42.9%)	8 (57.1%)	0	2	0 (0%)	2 (100%)	0
Spain	7571	992 (14.4%)	5882 (85.6%)	697	459	56 (13.6%)	356 (86.4%)	47
United Kingdom	3704	75 (52.1%)	69 (47.9%)	3560	139	47 (45.6%)	56 (54.4%)	36
Sweden	260	89 (34.8%)	167 (65.2%)	4	37	10 (27.8%)	26 (72.2%)	1
Switzerland	554	0	0	554	25	0	0	25

Countries which reported cases in period 2 shown only. [a]Travel history outside the country of notification in the 21 days prior to onset of symptoms. Percentages calculated for known information.
